# Role of Bowel Preparation in Adenoma Detection Rate and Follow-up Recommendations in African American Dominant Patient Population

**DOI:** 10.7759/cureus.16065

**Published:** 2021-06-30

**Authors:** Hamid-Reza Moein, Eskara Pervez, Salina Faidhalla, Heba Habbal, Hajra Khan, Anshu Wadehra, Mahvish Khalid, Diana Kakos, Paul Naylor, Bashar Mohamad

**Affiliations:** 1 Internal Medicine, Sinai-Grace Hospital/Detroit Medical Center, Detroit, USA; 2 Internal Medicine, Wayne State University/Detroit Medical Center, Detroit, USA; 3 Internal Medicine, Beaumont Hospital, Dearborn, USA; 4 Internal Medicine, Wayne State University School of Medicine, Bloomfield Hills, USA; 5 Gastroenterology, Wayne State University School of Medicine, Detroit, USA; 6 Gastroenterology and Hepatology, Wayne State University, Detroit, USA

**Keywords:** bowel preparation quality, adenoma detection rate, surveillance colonoscopy recommendations, screening colonoscopy, surveillance colonoscopy

## Abstract

Introduction: Bowel preparation quality in colonoscopy is one of the most essential components of quality assessment. According to the latest guidelines, inadequate bowel preparation warrants repeat colonoscopy in less than a year. Our aim was to investigate the role of bowel preparation in adenoma detection rate (ADR), its relationship with patients' demographics, and compliance with subsequent surveillance recommendations with guidelines.

Methods: This is a retrospective chart review study. Bowel preparation quality was divided into three categories: high, intermediate, and low. ADR and polyp detection rates (PDR) were calculated as the number of patients with adenoma or polyp divided by the total number of patients.

Results: Among 1,062 patients (934 African American and 128 non-African American) 81%, 11%, and 8% had high, intermediate, and low-quality bowel preparations, respectively. Race, gender, age, type of endoscopist, and body mass index did not play any role in bowel preparation quality. ADR and PDR were significantly higher in African Americans as compared to non-African Americans. ADR was significantly lower in the low-quality as compared to the high- and intermediate-quality bowel preparations (OR=2.13; p=0.0032). Bowel preparation quality was not correlated with subsequent follow-up recommendations. Academic gastroenterologists and surgeons had the highest and lowest compliance with surveillance guidelines, respectively.

Conclusions: Racial and gender disparity appears to have no meaningful effect on the quality of bowel preparation. Only two categories (adequate [high/intermediate] or inadequate [low-quality]) may be used for follow-up recommendations. Non-compliance with surveillance guidelines is concerning and may inadvertently increase the interval risk of colorectal cancer.

## Introduction

Colonoscopy is currently the gold standard of colorectal cancer (CRC) detection and also is used to prevent or reduce the risk of CRC by removing adenomatous polyps [1]. Quality metrics of screening and surveillance colonoscopies should be monitored routinely. Bowel preparation and adenoma detection rate (ADR) are the most important quality measures in colonoscopy [2,3]. Bowel preparation quality plays a crucial role in diagnostic accuracy and therapeutic safety of colonoscopy [3-5]. Inadequate bowel preparation can result in an estimated adenoma miss rate of 28%-42%, prolonged procedure times, increased costs, and also increased adverse events [3,4]. According to the latest Standards of Practice Committee of the American Society for Gastrointestinal Endoscopy, in patients with inadequate bowel preparations repeat colonoscopy in less than a year is recommended [6].

Studies showed conflicting results regarding the correlation of bowel preparation quality and ADR [5,7-11]. The majority of studies demonstrated similar ADR among intermediate and high-quality bowel preps and significantly lower ADR in low-quality bowel preps [8]. Some studies did not find any correlation between ADR and bowel preparation [10-12]. Moreover, better bowel preparation is not always equal to better ADR [9].

Information in African American patient population regarding bowel preparation and ADR is lacking in the literature. Knowing that African Americans have the highest prevalence of colorectal adenoma [13] and the highest incidence and mortality rate from CRC [14], characterizing factors contributing to bowel preparation quality and its correlation with ADR are highly important in this patient population. Therefore, we aimed to investigate the correlation between bowel preparation and ADR in African American population and compare it to non-African Americans. Considering the well-known effect of body weight, gender, and age on ADR [10,11], we evaluated the relationship of these risk factors with bowel preparation. Furthermore, we sought to investigate the adherence of endoscopists to gastrointestinal society guidelines for follow-up colonoscopy recommendations.

## Materials and methods

Study population and measurements

This is a retrospective, cross-sectional, chart review study. Consecutive electronic medical records of patients who underwent screening or surveillance colonoscopy at Harper Hospital/Detroit Medical Center’s endoscopy suite (open access) in the last six months of 2017 were reviewed. Age, gender, body mass index (BMI), race, bowel preparation quality, number of polyps, adenoma detection, endoscopist specialty (Gastroenterologist vs. surgery), surveillance vs. screening colonoscopy, and follow-up recommendations were reviewed from electronic medical records. This study was approved by the Institutional Review Board of Wayne State University.

Bowel preparation

Drinking four liters of polyethylene glycol the day before colonoscopy was used as the bowel preparation regimen in all the patients. Bowel preparation quality was recorded based on physician reporting in the procedure notes. Most endoscopists used a modified Aronchick bowel preparation scale to describe the bowel preparation quality [15]. In brief, excellent quality is defined as >95% of the mucosal surface seen; good quality is defined as a >90% of the mucosal surface seen with a large volume of clear; fair quality is defined as some semisolid stool that could be suctioned or washed away but >90% of the mucosal surface seen; poor quality indicates <90% of the mucosal surface seen with semisolid stool that could not be suctioned or washed away; and insufficient quality indicates that fecal material could not be cleared.

Only one patient had a report based on Boston Bowel Preparation Scale, which was >5. That was considered equal to good prep on the Aronchick scale, according to a validation study [16]. For analysis purposes in this study, we divided the bowel preparations into three different groups: high quality (including excellent and good preparation), intermediate quality (fair preparation), and low quality (poor/insufficient preparation). In 12.9% of colonoscopies, the bowel preparation quality was not reported by physicians and in those cases, a category was chosen indirectly based on the other components of the endoscopy report such as comments related to visualization of the colon, small polyp detection, and follow-up recommendations.

Colonoscopy and follow-up recommendations

All colonoscopies were performed by certified academic and non-academic gastroenterologists (non-AGI) or surgeons. The follow-up interval recommendation for the next colonoscopy is obtained from the procedure notes written by endoscopists after their procedure. Then the follow-up recommendations were compared with the relevant available guidelines in 2017 based on the number of polyps or adenomas and quality of preparation [4,6,17]. In brief, guidelines indicate follow-up colonoscopy in less than a year for inadequate bowel preparation, three to five years for adenomas based on their number and size, and 10 years for hyperplastic or benign polyps.

ADR and PDR calculation

ADR was defined as the proportion of colonoscopies with at least one adenoma detected and is reported separately for each bowel preparation. Polyp detection rate (PDR) was calculated similarly as the number of examinations with at least one polyp was divided by the total number of examinations.

Statistical analysis

Statistical analysis was performed by JMP 14.0 Software (SAS Institute Inc., Cary, NC, USA). Descriptive data are presented as mean ± SEM. Odds ratio (ORs) and 95% confidence intervals (CIs) were preferentially used for the comparisons of ADR and PDR. ANOVA and Chi-square tests were used to compare the quality of bowel preparation with other variables. Linear regression and Spearman test were used for correlations between bowel preparation and ADR with demographics. A P-value of <0.05 is considered significant. 

## Results

A total of 1,062 patients were included and analyzed in this study out of 1,095 reviewed electronic charts. Among those 33 excluded were six with a tumor, 13 with missing pathology or lost biopsy and 14 were aborted. The predominant study population were African Americans who constituted 87.9% of the patients. Non-African American population included Caucasians (8.5%), Asians (1.6%), Hispanics (0.84%), American Indians (0.75%), and Middle Eastern (0.18%). The mean age was 59 ± 0.19 years and 46.6% were males. African American patients were statistically younger and had higher BMI as compared to non-African Americans (Table 1).

**Table 1:** Demographics and bowel preparation quality among African American and non-African American patients. BMI, Body mass index. *P-value comparing African Americas vs. non-African Americans using t-test or Chi-square tests.

	African American (n=934)	Non-African American (n=128)	Total (n=1,062)	P*
Age (year)	59 ± 0.2	60 ± 0.6	59 ± 0.2	0.042
Gender (male %)	46%	45%	54%	0.91
BMI (kg/m^2^)	31 ± 0.2	30 ± 0.7	31 ± 0.2	0.02
Colonoscopy indication (screening %)	80%	74%	80%	0.10
Bowel preparation quality				
High (Excellent/Good)	747 (82%)	108 (84%)	855 (81%)	0.4
Intermediate (Fair)	107 (12%)	10 (8%)	117 (11%)	0.43
Low (Poor/Insufficient)	80 (8%)	10 (8%)	90 (8%)	0.43

The majority of colonoscopies were performed by AGI (66%) followed by non-AGI/private gastroenterologists (17%) and surgeons (17%).

Bowel preparation quality and demographics

Bowel preparation quality is reported separately for African Americans and non-African Americans in Table 1. In total, 81%, 11%, and 8% of patients had high, intermediate, and low-quality bowel preparations, respectively. There was no significant difference between African Americans and non-African Americans in respect to bowel preparation quality (Table 1). The role of demographics such as age, gender, and BMI that may play a role in the quality of bowel preparation was evaluated. Demographics of patients with different bowel preparation categories were similar (Table 2).

**Table 2:** Correlation of bowel preparation quality with demographics among African Americans and non-African Americans who had similar bowel preparation regimen. BMI, Body mass index. *P-values representative of the difference between groups in each column.

Bowel preparation quality	African American			Non-African Americans		
	Age	Male (%)	BMI (kg/m^2^)	Age	Male (%)	BMI (kg/m^2^)
High	58.3 ± 0.2	46%	31 ± 0.27	59 ± 0.16	46%	29.0 ± 0.7
Intermediate	59.4 ± 0.5	51%	31 ± 0.73	63.2 ± 2.14	30	31.5 ± 2.3
Low	59.2 ± 0.6	50%	32 ± 0.84	61 ± 2.14	50%	28.7 ± 2.3
P*	0.17	0.31	0.73	0.16	0.58	0.62

Colonoscopy metrics are reported in Table 3. Optimal performance as defined by reaching the cecum was significantly lower in the low-quality bowel preparation as compared to the intermediate- and high-quality bowel preparations (p=0.0001). Total mean number of found polyps were 1.2 ± 0.0, 1.2 ± 0.1, and 0.7 ± 0.1 in high, intermediate, and low-quality bowel preparations, respectively (p=0.028). The total PDR was significantly higher in high- and intermediate-quality bowel preparation as compared to low-quality bowel preparations (p=0.0005). Similarly, ADR was significantly higher in high- and intermediate-quality bowel preparations in comparison with low-quality bowel preparation (p=0.01 for both, respectively) (Table 3). ADR and PDR were similar in high- vs. intermediate-quality bowel preparation (p>0.05) (Table 3). ADR was significantly higher in African American patients (38%) compared with non-African American patients (27%) (p=0.0213). Similarly, PDR was also higher in African Americans compared to non-African Americans (56% vs. 46%; p=0.0310).

**Table 3:** Colonoscopy metrics as a function of bowel preparation quality. ADR, Adenoma detection rate. *P values for columns calculated based on Pearson chi-square test.

Bowel preparation quality	Cecum intubation (n=1,062)	Polyp detection rate (n=1,062)	Adenoma detection rate (n=1,062)	ADR in screening colonoscopies (n=846)	ADR in surveillance colonoscopies (n=216)
High	99%	58%	38%	37%	44%
Intermediate	98%	49%	38%	33%	52%
Low	86%	38%	22%	18%	39%
P*	0.0001	0.0005	0.013	0.0078	0.65

Follow-up recommendations and compliance of endoscopists with standard guidelines

Among patients with low-quality bowel preparation (87 patients), 21.0% did not have any follow-up recommendations (Table 4). When documented follow-up recommendations for low-quality bowel preparation were evaluated by specialty, AGI had more documentation (93%) as compared to non-AGI (57%) or surgeons (39%) (p=0.0001). Surgeons had the lowest compliance with standard guidelines (22%) as compared to non-AGI (59%) and AGI (80%) (p=0.0001). Participation of fellows with AGI did not lead to a significant change in the percentage of follow-up recommendation documentation (93%) or correct recommendations (80%) as compared to the AGI alone (93% and 80%, respectively). We did not find any significant correlation between different group of endoscopists and bowel preparation quality (p=0.12).

**Table 4:** Guideline-directed follow-up recommendations based on the bowel preparation quality. *Recommendations are based on Standards of Practice Committee of the American Society for Gastrointestinal Endoscopy published in 2015 [4].

Bowel preparation quality	Recommendation* is provided (%)	Correct recommendation (%)	Longer recommendation (%)	Shorter recommendation (%)
High	78%	73%	2%	25%
Intermediate	83%	68%	9%	23%
Low	79%	78%	18%	4%

There was no significant correlation between different bowel preparation categories and provided recommendations or recommendation compliance rates (Table 4). The most non-compliance with follow-up guidelines was observed in the intermediate-quality bowel preparation (32%) as compared to low and high-quality bowel preparations (22% and 27%, respectively). This could reflect possible ambiguity in this small number of patients with respect to physician decision. Given that poor preparation quality should require a return within a year when a recommendation was given, 18% of the time it was for a longer interval than recommended by the guidelines (Table 4).

## Discussion

Bowel preparation is one of the essential quality metrics in colonoscopy [2,3]. In this study, we specifically investigated bowel preparation and its role in detecting polyps and adenomas among African American dominant patient population, which data are limited. We found that adequate (high and intermediate) bowel preparation results in higher ADR as compared to low-quality bowel preparation (38% vs. 22%, OR=2.13; p=0.0032). The ADR gap between high- and intermediate- bowel preparation vs. low-quality bowel preparation was even higher in the screening colonoscopies (36% vs. 19%, OR=2.54; p=0.0023). This is very important since ADR has a reverse correlation with CRC detection and death [2,18-20]. Similar to our study, Clark et al. [8] demonstrated that high- and intermediate-quality bowel preparations resulted in higher ADR as compared to low-quality bowel preparation (OR=1.39). In addition, we did not find any significant difference in ADR among high- and intermediate (fair)-quality bowel preparation, which was similar to Clark et al. [8]. However, Menees et al. [21] demonstrated a 28% adenoma miss rate in those patients with fair (intermediate-quality) bowel preparation when repeated colonoscopy in a three-year period. On the other hand, Park et al. [11] and Rai et al. [10] did not find any significant correlation between the quality of bowel preparation and ADR. Nevertheless, they found a trend and they attributed their finding to possibly nonsufficient sample size to detect the difference. Analysis from New Hampshire Colonoscopy Registry also did not find any significant correlation between different qualities of bowel preparation and ADR, which was consistent for both screening and surveillance colonoscopies [12]. In the same line, despite finding a trend towards lower ADR in surveillance colonoscopies with low-quality bowel preparation compared to adequate bowel preparation, there was no significant difference. This finding can be attributed to a low number of surveillance colonoscopies in this study (20% of total colonoscopies) or longer withdrawal time in surveillance vs. screening colonoscopies (not recorded in this study). In addition, we showed significantly higher ADR and PDR in African Americans in comparison with non-African Americans. Similarly, Lebwohl et al. demonstrated significantly higher ADR and prevalence of adenoma and advanced adenoma in African Americans in comparison with Caucasians [13]. However, a recent review indicates mixed evidence in ADR and prevalence of adenomas among African Americans and Caucasians [22].

Inadequate bowel preparation has shown increased total cost of a colonoscopy, increased procedure time and adenoma missed rate [3,4]. Our data suggest that race itself does not play a role in the quality of bowel preparation. This is in accordance with Lebwohl et al. in which they did not find any significant difference in bowel preparation quality among different races after multivariant analysis [23]. On the other hand, Appannagari et al. reported African American race as an independent risk factor for inadequate bowel preparation [24]. They found 50% increased risk of inadequate bowel preparation among African Americas as compared to whites despite controlling for education and income. Despite the statistically significant difference in age and BMI between African Americans and non-African Americans, in our study, gender, age, endoscopist’s specialty, and BMI were not significantly correlated with bowel preparation quality. On contrary, other studies showed that obesity, advanced age, and male gender could be risk factors of poor bowel preparation [10,11,23,25,26]. However, Anderson et al. showed a significant correlation with younger age and poor bowel preparation [12]. We did not observe any significant difference in bowel preparation quality among different type of endoscopists (i.e., surgeons, AGI, and non-AGI). However, Sapci et al. demonstrated that lower bowel preparation quality among surgeons had a correlation with the lower ADR in that group of endoscopists in comparison with gastroenterologists [27]. One of the reasons for discrepancies between different studies and our study can be different patient populations and races. This study is comprised of African American dominant population but the above-mentioned studies [10-12,23-26] were either Caucasian or Asian dominant. Poor compliance with bowel preparation instructions, comorbidities such as diabetes mellitus and stroke, longer appointment wait times [4,25,26], smoking [11,12], procedure time (after 11 AM [23] or after 12 PM [24]), Medicaid insurers [23], and single status [23] were all have been associated with low-quality bowel preparation.

Follow-up colonoscopy recommendations in patients with low-quality bowel preparation were missing in 24.2% and were not compliant with guidelines in 16.7% of cases in our study. Menees et al. reported that only 13% of their endoscopists complied with current recommended guidelines [21]. On the other hand, Walker et al. showed 97% compliance of a single-center endoscopy suite with follow-up colonoscopy guidelines [28]. Reasons for non-compliance for guidelines include knowledge gap, not agreeing with the current national guidelines, or just simply because it has not been recorded in the electronic records by the endoscopists. A national survey result indicates a knowledge gap among gastroenterologists about the current surveillance guidelines [29]. This survey in 2004 showed that only 78% were familiar with the current national guidelines for surveillance colonoscopy and up to 76% disagreed with the guidelines [29]. A more updated and comprehensive investigation is warranted to evaluate the reasons for non-compliance of gastroenterologists or surgeons with national guidelines. 

One of the strengths of this study is that all the included patients used the same bowel preparation regimen. This helped in making the comparisons more accurate and also allowed us to investigate other risk factors in bowel preparation quality with less bias. Moreover, this study is unique because of the African American dominant patient population, a population in which the current literature is limited. Our study had some limitations. Non-African American patient population sample size was smaller than African Americans, which might have decreased the power for detection of any difference among these two groups. Due to a limited number of patients of other races including Asian and Hispanic, we combined Caucasians and other races except for African American into one group of non-African American. In addition, 12.9% of endoscopists did not record the bowel preparation quality and in those cases, we concluded the bowel prep quality based on their findings and follow-up recommendations, which might not have been exactly accurate. Patients’ compliance with bowel preparation instructions and follow-up recommendations, socioeconomic status, education, marital status, insurance, and time of the endoscopy (which were reported in some studies as risk factors for suboptimal bowel preparation) were not recorded in this study. Our results are limited to out-patient setting and in an African American dominant patient population and may not be generalizable.

## Conclusions

In conclusion, two categories of bowel preparation (i.e., adequate or inadequate) appear relevant for follow up recommendations after colonoscopy. The current study indicates that only inadequate or low-quality bowel preparation was associated with lower ADR. We did not observe any significant correlation between age, gender, race, and BMI with bowel preparation quality in this African American dominant patient population. Large prospective studies in African Americans are warranted to untangle the main risk factors for low-quality bowel preparation in this patient population and to address the conflicting results. Important quality measures such as recording follow-up recommendations and guideline-appropriate recommendations should be emphasized as when these measures are missing the risk of interval CRC may increase.
